# Prof. Powell’s Lecture

**Published:** 1883-12-20

**Authors:** 


### *PROF. POWELL'S LECTURE.*

The profession will doubtless read with interest Prof. Powell's opening lecture made to the class of the Southern Medical College at the present session.

The fitness of Atlanta as a medical centre for the South is, we think, conclusively shown, and also the ample facilities furnished by the Southern Medical College for a high order of medical education. The important points also, that correct principle and moral training are not disregarded by the Trustees and Faculty of this school will be favorably noted by the medical profession, and by all who desire to send their sons to a medical institution.

There were many visitors present when the lecture was delivered, and it was by a resolution of a prominent citizen, seconded by the class, and carried by a unanimous vote that the lecture was published. W.

